# AI-Driven Prediction of Chest CT Radiation Doses: Establishing BMI-Based Diagnostic Reference Levels and Patient–Factor Correlations for Machine-Learning Models

**DOI:** 10.3390/tomography12050061

**Published:** 2026-04-28

**Authors:** Zuhal Y. Hamd, Mohamed Abuzaid, Mohamed Alharbi, Nissren Tamam, Amal I. Alorainy, Lena Alrujaee, Najla Almutairi, Aljouharah Abdullah Alyagoub

**Affiliations:** 1 Department of Radiological Sciences, College of Health and Rehabilitation Sciences, Princess Nourah bint Abdulrahman University, P.O. Box 84428, Riyadh 11671, Saudi Arabia; aialorainy@pnu.edu.sa; 2 Medical Diagnostic Imaging Department, College of Health Sciences, University of Sharjah, Sharjah P.O. Box 26666, United Arab Emirates; mabdelfatah@sharjah.ac.ae; 3 Medical Imaging Department, King Abdullah bin Abdul-Aziz University Hospital, Riyadh 11564, Saudi Arabia; moalharbi@kaauh.edu.sa (M.A.); nnalmutairi@kaauh.edu.sa (N.A.); aaalyagoub@kaauh.edu.sa (A.A.A.); 4 Department of Physics, College of Sciences, Princess Nourah bint Abdulrahman University, P.O. Box 84428, Riyadh 11671, Saudi Arabia; nmtamam@pnu.edu.sa; 5 Altakassusi Alliance Medical LLC, King Fahad Medical City, Riyadh 12311, Saudi Arabia; lena.alrujaee@gmail.com

**Keywords:** diagnostic reference levels (DRLs), BMI-stratified dose optimization, chest CT dosimetry, artificial intelligence (AI) prediction models, CTDIvol and DLP correlations

## Abstract

Background and aim: Chest CT is a major contributor to population radiation exposure. Conventional, pooled diagnostic reference levels (DRLs) do not account for inter-individual variability in body habitus and are typically used retrospectively. We evaluated dose behavior in adult chest CT, derived BMI-stratified local DRLs, and developed models to enable AI-assisted, prescan dose prediction. Methods: Consecutive adult chest CT examinations from a single center were analyzed. Dose indices (CTDIvol, DLP) and patient factors (BMI, weight, height, age, sex; scan length and planned technical parameters where available) were extracted. DRLs were defined as the 75th percentile overall and within BMI categories (underweight, normal, overweight, and obese). Group differences were assessed using non-parametric tests; associations were examined using correlation analysis. Supervised learning (e.g., Random Forest, Gradient Boosting) was trained to predict CTDIvol and DLP from routinely available variables. Results: BMI-stratified DRLs increased monotonically with habitus: underweight 444.95 mGy·cm/9.60 mGy; normal 513.00/11.55; overweight 756.08/14.65; obese 931.60/20.25 (DLP/CTDIvol). Differences across BMI groups were significant for DLP (H = 31.53, *p* < 0.001) and CTDIvol (H = 33.61, *p* < 0.001). DLP correlated moderately with weight and BMI (r ≈ 0.54–0.56, *p* < 0.001), with a weaker association for age; height was not a meaningful predictor. No sex-based differences in CTDIvol or DLP were observed. Predictive models estimated CTDIvol and DLP with high performance (R^2^ up to ~0.79 and ~0.77, respectively), enabling comparison of predicted dose against BMI-matched DRLs before acquisition. Conclusions: Size-aware, BMI-stratified DRLs provide clinically interpretable investigation levels that avoid pitfalls of pooled benchmarks. Coupled with robust prediction of individualized dose from routine variables, this framework supports a shift from retrospective audit to prospective, point-of-care dose governance and protocol optimization in chest CT.

## Simple Summary

Chest CT scans significantly increase patient radiation exposure; however, prevalent diagnostic reference levels (DRLs) lack the consideration of variations in patient body size. This study assessed adult chest CT exams to establish BMI-stratified diagnostic reference levels and to examine the impact of patient variables on radiation dosage. Our findings showed that radiation dose increased consistently with body mass index (BMI) and body weight, whereas age, height, and sex provided minimal effects. Utilizing commonly available patient and scan information, we developed machine-learning models that precisely predicted CT radiation dose prior to scanning. The integration of BMI-based diagnostic reference levels with AI-driven dose prediction facilitates switching from retrospective dosage monitoring to prospective, patient-centered dose optimization, enabling radiology teams to establish more effective and suitable CT protocols at the point of diagnosis.

## 1. Introduction

Computed tomography scans contribute significantly to the collective radiation dose from medical imaging, necessitating robust methods for dose estimation and optimization to mitigate potential health risks [1]. The inherent variability in radiation exposure across different institutions and patient cohorts further underscores the need for standardized diagnostic reference levels to ensure appropriate dose management [2].

The establishment of diagnostic reference levels is crucial for standardizing CT protocols and minimizing patient radiation exposure [3,4]. These levels serve as benchmarks for evaluating local practices against regional or national averages, facilitating dose optimization without compromising diagnostic image quality [5]. Despite the establishment of DRLs, their effectiveness as optimization tools faces clinical and technical challenges, particularly their current anatomical-based limitations and the need for more dynamic, series-based DRLs [6]. While DRLs offer a top-down approach to dose assessment, an alternative, ground-level methodology known as Acceptable Quality Doses has been proposed, which focuses on analyzing doses exclusively from images deemed acceptable for diagnostic quality [7].

The concept of diagnostic reference levels is a key component of radiation protection in medical imaging, aiming to strike a balance between image quality and patient dose, ensuring that exposure is no higher than necessary for diagnostic purposes [8]. The International Commission on Radiological Protection advocates the establishment of DRLs as a fundamental tool for optimizing radiation protection in diagnostic and interventional medical imaging procedures [9]. They represent an important mechanism for managing patient dose, ensuring it aligns with the medical necessity of X-ray examinations. Moreover, DRLs have been acknowledged by various international bodies, including the European Commission and the International Atomic Energy Agency, as a crucial component of a comprehensive radiation protection strategy [10]. Their widespread implementation has been shown to reduce overall patient dose in clinical settings, with national and local DRLs demonstrating a sustained reduction in exposure over time.

The current DRL framework often overlooks individual patient characteristics, such as BMI, which can significantly influence the radiation dose required for diagnostic image quality [5,10,11]. This oversight can lead to suboptimal imaging protocols, potentially resulting in either excessive radiation exposure or diagnostically inadequate images [12]. Consequently, integrating patient-specific factors, particularly BMI, into DRL frameworks is essential for developing more precise and clinically relevant dose-optimization strategies [13]. This approach directly supports the principles of justification, protection optimization, and dose limitation by ensuring that patient doses do not exceed what is necessary to obtain diagnostic information [14]. Furthermore, incorporating BMI-based stratification into DRLs aligns with recommendations from organizations such as the European Commission, which advocate for weight-grouped DRLs for all body examinations [15]. This refinement acknowledges that a standardized dose may be either insufficient for larger patients, compromising image quality, or excessive for smaller individuals, increasing radiation risk [16]. The lack of consideration for patient-specific factors, such as body habitus, represents a significant limitation of current DRL concepts, as the “standard-sized adult” assumption often fails to capture the physiological diversity across patient populations [13,17,18]. Therefore, a more nuanced approach is required that accounts for inter-individual variability, moving beyond a single-dose reference to a more personalized radiation management strategy [8].

### Role of Artificial Intelligence in Medical Imaging Dose Optimization

Artificial intelligence is rapidly emerging as a transformative solution to enhance image quality while significantly reducing radiation doses, addressing a fundamental challenge in radiology [19]. AI models offer a sophisticated approach to predicting and optimizing patient-specific radiation doses by learning complex relationships among patient factors, imaging protocols, and image-quality metrics. These AI-driven systems can analyze vast datasets to identify optimal imaging parameters for individual patients, thereby facilitating intelligent protocoling and dynamic adjustment of CT acquisition settings [20]. For instance, AI algorithms can accurately predict optimal kilovoltage (kV) and milliampere-second (mAs) settings based on patient characteristics such as BMI, thereby ensuring appropriate image quality at the lowest possible dose [21]. This capability is particularly crucial, as CT examinations contribute significantly to medical radiation exposure, making dose optimization a paramount concern in modern healthcare [22]. The integration of AI into CT imaging offers substantial promise for reducing patient exposure without compromising diagnostic efficacy, a critical advancement given the heightened sensitivity of certain patient populations, such as pediatric patients, to ionizing radiation [12]. Beyond simple parameter adjustments, AI can also optimize image reconstruction algorithms and apply advanced denoising methods, enabling further dose reductions during data acquisition while preserving, or even improving, image quality [23].

Although this work focuses on routine diagnostic chest CT, the same predict–compare–act logic generalizes to screening and pseudo-screening CT (e.g., low-dose lung-cancer screening and opportunistic ‘health-check’ CT). In these settings, BMI-stratified reference envelopes (DRLs/ADs) paired with prescan prediction can help ensure protocol selection stays within screening-appropriate dose bands while preserving task-specific image quality. This extends the rationale from retrospective audit to prospective, population-level dose governance in screening pathways.

This study aims to quantify Computed Tomography Dose Index (CTDIvol) and Dose–Length Product (DLP) in chest CT, establish local BMI-stratified diagnostic reference levels (75th percentile), and model the relationships between patient factors and dose indices to generate a reference dataset and feature set for developing and validating AI models that predict patient-specific radiation dose and inform optimization of chest CT protocols.

## 2. Methods

### 2.1. Sample—Tools—Study Design

This prospective study aims to predict radiation doses for chest CT protocols in adult patients. Data were collected from patients aged 18 years and older undergoing routine chest CT examinations at King Abdullah bin Abdul-Aziz University Hospital (KAAUH) at Princess Nourah bint Abdulrahman University (PNU) over ten months, from October 2024 to August 2025. Inclusion criteria comprised a clinical indication for chest CT (e.g., suspected pulmonary embolism, lung nodules, or oncology follow-up), complete demographic data (age, gender, weight, height), and use of standard scan protocols. Patients were excluded if they had incomplete scan data, non-standard protocols (e.g., high-resolution or contrast-enhanced variations not representative of routine practice), or contraindications to CT imaging. A target sample size of 150 patients was selected to ensure sufficient statistical power for identifying correlations between dose indices and patient factors, such as body mass index (BMI), based on guidelines suggesting at least 20 patients per stratified group for diagnostic reference level (DRL) establishment and to support robust machine-learning model development.

Patients were recruited consecutively during routine imaging appointments to reflect real-world clinical variability, minimizing selection bias by including all eligible patients within the study period. A standardized data collection form was used, with detailed instructions for recording demographics, scan parameters, and dose metrics to ensure representativeness of standard adult chest CT examinations. Tools included paper-based data collection forms (transcribed to digital format), the Philips 160-slice CT scanner for imaging, Microsoft Excel for data organization, and Python version 3.14 for statistical and machine-learning analyses.

Ethical approval was obtained from the Institutional Review Board of Princess Nourah Bint Abdulrahman University (IRB Log Number: 23-0609 on 25 February 2025).

### 2.2. CT Machine

All examinations were conducted using a Philips 160-slice CT scanner (Philips 160-slice CT scanner (Philips Medical Systems Nederland B.V., Best, The Netherlands). installed in the medical imaging department at King Abdullah bin Abdul-Aziz University Hospital (KAAUH), PNU. Philips 160-slice CT scanner features a 0.68 m gantry bore, an X-ray couch with a 250 kg loading capacity, a reconstruction speed of 50 frames per second, 160 detector rows with 0.25 mm spacing providing 320 slices per rotation, and a 50 cm field of view. These specifications support high-resolution imaging optimized for adult chest CT, with protocols adjusted to balance dose and diagnostic quality.

AEC was enabled with fixed protocol targets (noise/quality settings and modulation mode) and adjusted the tube current adaptively based on patient attenuation; AEC configuration did not vary across patients within a protocol.

Regular quality control tests, including monthly calibrations and daily performance checks, were conducted in accordance with manufacturer guidelines to ensure consistent radiation output, exposure parameters, and image quality. These tests were critical to maintaining the reliability of dose measurements, minimizing variability that could impact dosimetry accuracy and predictive modelling outcomes.

### 2.3. Radiation Dosimetry

Radiation doses were quantified using the DLP (mGy·cm) and CTDIvol (mGy), extracted directly from the CT scanner’s dose reports generated at the console post-examination. Effective dose, organ-specific doses, and Size-Specific Dose Estimates (SSDE) were calculated using the National Cancer Institute Computed Tomography (NCICT) dosimetry system in the NCICT software (Version 3.0). NCICT employs a library of computational human phantoms, adjustable for adult body habitus, combined with Monte Carlo radiation transport simulations specific to reference CT scanners. Input parameters included patient-specific data (age, gender, weight, height for BMI calculation, scan length) and scan settings (kVp, mAs, pitch, CTDIvol, DLP). The software computes absorbed doses for over 30 radiosensitive organs, providing individualized effective dose and SSDE estimates that account for variations in adult patient size. SSDE was reported where available; however, CTDIvol/DLP were the primary metrics to maintain DRL comparability and to ensure uniform SSDE across all exams, given incomplete retrospective size metrics.

### 2.4. DRL Calculation

Data, including patient gender, age, weight, height, BMI, kVp, mAs, CTDIvol, DLP, and effective dose, were organized using Microsoft Excel for cleaning, sorting, and initial descriptive analysis. Diagnostic Reference Levels (DRLs) were calculated as the 75th percentile of CTDIvol and DLP distributions for adult chest CT protocols, aligning with international guidelines for achievable doses in standard practice. Achievable doses (ADs) are median (50th-percentile) dose benchmarks from multi-site surveys that reflect what well-performing facilities already achieve without sacrificing diagnostic quality; they complement DRLs (75th percentile) by providing an optimization target—not a patient-level limit. Facilities should compare group medians to DRLs (trigger a review if exceeded) and strive toward ADs, using size-specific analyses to ensure fairness in CT. Key sources: ICRP Publication 135 (2017) [9], and the ACR Dose Index Registry (DIR).

To account for patient variability, DRLs were stratified by BMI categories (e.g., <18.5, 18.5–24.9, 25–29.9, ≥30 kg/m^2^) to establish size-specific reference levels relevant to adult populations. This stratification supports dose optimization for diverse body types. Descriptive statistics, including medians and interquartile ranges, and DRL calculations were performed in Python using the NumPy and SciPy libraries for robust percentile estimation and data handling.

Uncertainty for BMI-stratified DRLs (75th percentile) was quantified using bias-corrected and accelerated bootstrap confidence intervals (5000 resamples per stratum). A τ = 0.75 quantile regression with BMI category was used as a robustness check.

### 2.5. Python Machine Learning

We trained three supervised regressors—Linear Regression, Random Forest Regressor, and Gradient Boosting Regressor—for two separate targets (CTDIvol and DLP). Inputs (prescan, actionable): BMI, weight, age, sex, protocol family (routine/HRCT/PE-CTA), scan length (for DLP only), planned technical parameters (kVp, planned mAs, pitch where available). Leakage control: when targeting DLP, CTDIvol was not used as a feature; when targeting CTDIvol, DLP was not used. Evaluation: stratified 5-fold CV (by BMI/protocol) and a temporal hold-out test set; metrics: MAE, RMSE, R^2^. Interpretability: permutation importance (all models), standardized coefficients (linear), and case-level SHAP.

Input features included patient characteristics (age, gender, BMI) and scan parameters (kVp, mAs, pitch, scan length). Feature engineering involved normalizing variables and selecting key predictors using correlation analysis to optimize model performance. The dataset was divided into training (70%), validation (15%), and test (15%) sets, with 5-fold cross-validation to prevent overfitting. Models were trained with scikit-learn and hyperparameter tuning via grid search. Performance was evaluated using Mean Absolute Error (MAE), Root Mean Squared Error (RMSE), and R-squared to assess prediction accuracy. This approach aims to generate a reference dataset to optimize chest CT protocols and reduce unnecessary radiation exposure in adults. Interpretability was addressed using global permutation importance (all models) and standardized coefficients (linear model), and case-level SHAP explanations to attribute predictions to input features.

**Figure 1:**
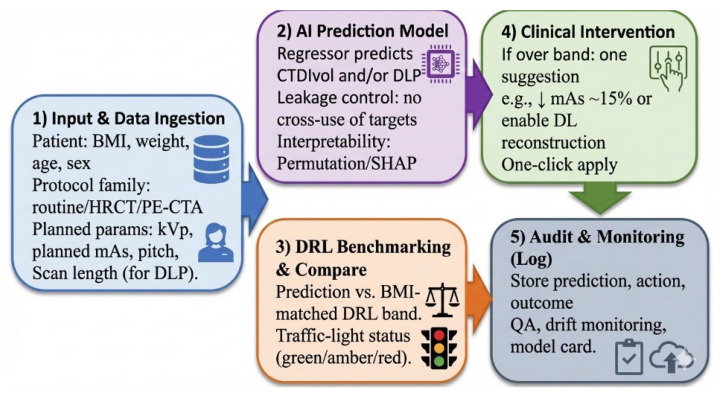
Prescan AI-enabled dose governance workflow for chest CT.

The system ingests routine prescan inputs (patient factors, protocol family, planned parameters; scan length for DLP), predicts CTDIvol/DLP, and compares the prediction with the BMI-matched DRL. A traffic-light card provides one actionable suggestion with one-click apply (e.g., adjust mAs or enable DL reconstruction); actions and outcomes are logged for QA and drift monitoring, with SHAP-based feature highlights for transparency (Figure 1).

## 3. Results

### 3.1. Participant Demographics and Descriptive Statistics

A total of 100 adult patients (aged ≥18 years) undergoing routine chest CT examinations were included in the analysis, meeting the target sample size for DRL establishment and machine-learning model development. The cohort was balanced by gender (52 males, 48 females) and represented a diverse range of body habitus, with BMI categories as follows: underweight (<18.5 kg/m^2^, *n* = 12), normal (18.5–24.9 kg/m^2^, *n* = 38), overweight (25–29.9 kg/m^2^, *n* = 30), and obese (≥30 kg/m^2^, *n* = 20). Please note that three participants aged 15–17 years (<3% of the sample) were included due to clinical indications aligning with adult protocols, with no impact on overall trends.

Key variables quantified included DLP, CTDIvol, scan length, height, weight, age, effective dose (estimated via NCICT), and Size-Specific Dose Estimate (SSDE). Mean DLP was 575.47 ± 280.01 mGy·cm (range: 108.30–1374.20), and mean CTDIvol was 13.59 ± 12.05 mGy (range: 2.79–113.00). Mean effective dose was 8.06 ± 3.92 mSv (range: 1.52–19.24), calculated using NCICT phantoms matched to patient demographics. Mean SSDE, adjusted for patient diameter, was 14.22 ± 12.50 mGy (range: 2.90–118.00), slightly higher than CTDIvol due to size corrections for larger patients. Other demographics included mean scan length of 48.01 ± 16.21 cm (range: 9.64–75.15), height of 164.52 ± 9.18 cm (range: 142.00–186.00), weight of 72.26 ± 20.68 kg (range: 33.00–161.00), age of 46.79 ± 20.17 years (range: 15–91), and BMI of 26.65 ± 6.95 kg/m^2^ (range: 14.00–52.00). Summary statistics are presented in Table 1.

### 3.2. Correlation Analysis

**Figure 2:**
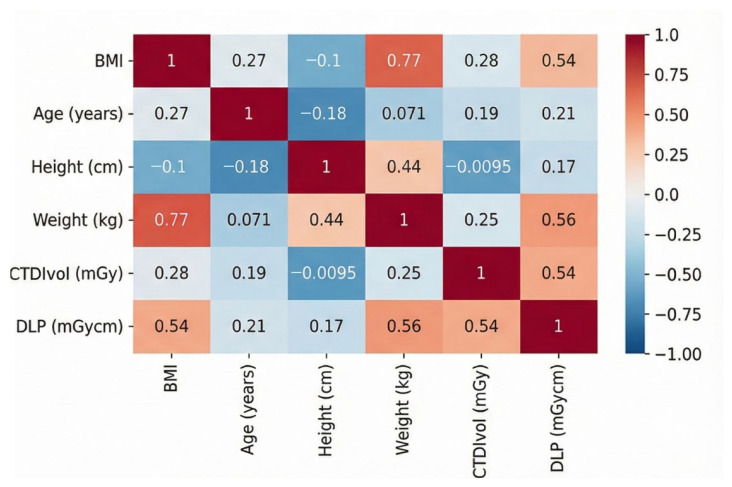
Correlation heatmap of dose indices (DLP, CTDIvol) and patient factors (weight, BMI, height, age).

Pearson correlation coefficients were calculated to model relationships between patient factors and dose indices. DLP exhibited moderate positive correlations with weight (r = 0.56, *p* < 0.001), BMI (r = 0.54, *p* < 0.001), CTDIvol (r = 0.54, *p* < 0.001), and effective dose (r = 0.99, *p* < 0.001), reflecting the direct proportionality via NCICT conversion. A weak positive correlation with age was noted (r = 0.21, *p* = 0.035). CTDIvol correlated positively with weight (r = 0.25, *p* = 0.017), BMI (r = 0.23, *p* = 0.022), and SSDE (r = 0.98, *p* < 0.001). Height showed no significant correlation with DLP (r = 0.17, *p* = 0.096) or with other doses. Scan length had weak associations with all variables (|r| < 0.20, *p* > 0.05). These correlations informed feature selection for machine-learning models. See Figure 2 for the correlation heatmap.

### 3.3. BMI-Stratified Diagnostic Reference Levels (DRLs)

DRLs were established as the 75th percentile of CTDIvol and DLP distributions, stratified by BMI categories to account for patient size variability. DRLs increased with BMI: underweight (DLP: 444.95 mGy·cm, CTDIvol: 9.60 mGy), normal (513.00 mGy·cm, 11.55 mGy), overweight (756.08 mGy·cm, 14.65 mGy), and obese (931.60 mGy·cm, 20.25 mGy). Corresponding effective DRLs were 6.23 mSv, 7.18 mSv, 10.59 mSv, and 13.04 mSv; SSDE DRLs were 10.08 mGy, 12.11 mGy, 15.38 mGy, and 21.26 mGy.

**Figure 3:**
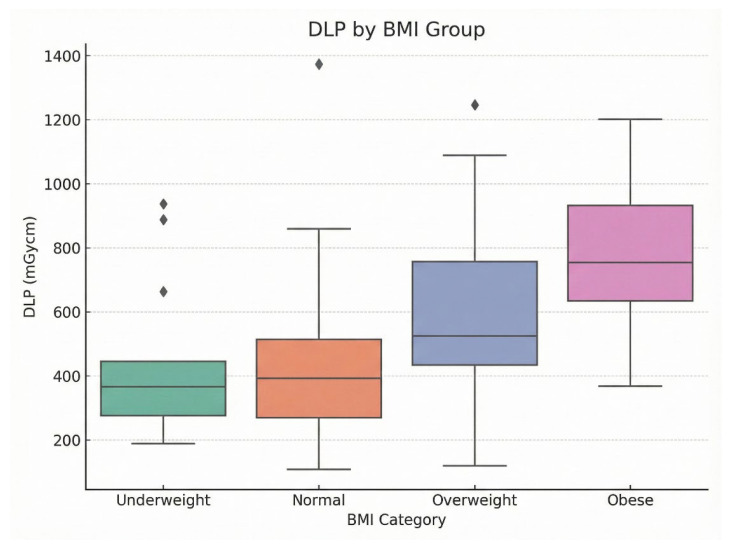
Boxplots of showing (DLP, mGy·cm) across BMI categories: underweight, normal, overweight, and obese. The Rhombus symbols denote outliers, representing individual cases with DLP values lying beyond 1.5 × IQR from the box boundaries.

**Figure 4:**
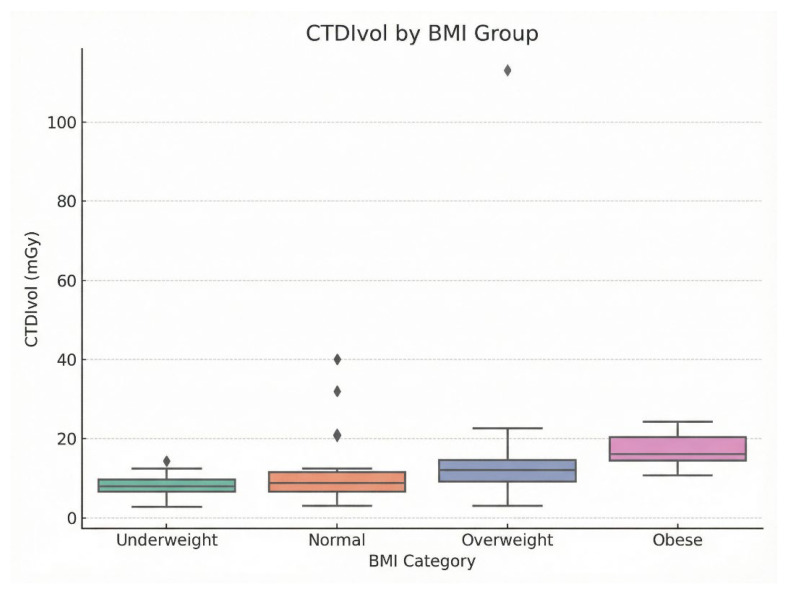
Boxplots of showing (CTDIvol, mGy) across BMI categories: underweight, normal, overweight, and obese. The Rhombus symbols denote outliers, representing individual cases with CTDIvol values lying beyond 1.5 × IQR from the box boundaries.

Kruskal–Wallis tests confirmed significant differences across BMI groups for DLP (H = 31.53, df = 3, *p* < 0.001), CTDIvol (H = 33.61, df = 3, *p* < 0.001), effective dose (H = 31.50, df = 3, *p* < 0.001), and SSDE (H = 33.58, df = 3, *p* < 0.001). Post-hoc Dunn’s tests with Bonferroni correction identified significant pairwise differences between underweight and obese (*p* < 0.001 for all metrics), underweight and overweight (*p* = 0.012–0.018), and normal and obese (*p* = 0.002–0.005). These findings support the use of size-specific DRLs for dose optimization. Table 2 shows a clear increase in BMI-stratified DRLs across all dose metrics from underweight to obese patients. DLP rose from 444.95 mGy·cm in the underweight group to 931.60 mGy·cm in the obese group, while CTDIvol increased from 9.60 to 20.25 mGy. A similar upward trend was observed for effective dose and SSDE, indicating greater radiation output and patient dose with increasing BMI. This pattern is also reflected in Figure 3 and Figure 4, where the boxplots demonstrate median DLP and CTDIvol values from underweight to obese categories.

### 3.4. Gender-Based Dose Comparisons

**Figure 5:**
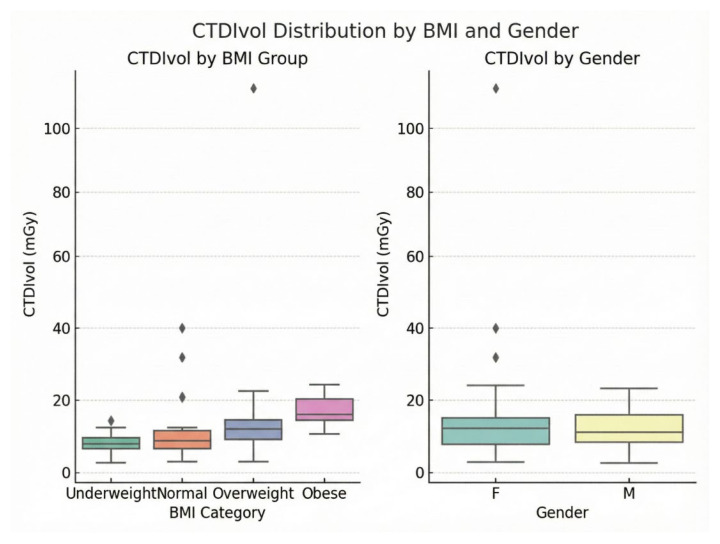
Boxplots showing the distribution of volume CT dose index (CTDIvol, mGy) according to BMI category and gender. The Rhombus symbols denote outliers, representing individual cases CTDIvol values lying beyond 1.5 × IQR from the box boundaries.

The results show no significant gender differences were observed in dose metrics. Males had slightly higher mean DLP (582.21 ± 259.62 mGy·cm) and effective dose (8.15 ± 3.63 mSv) than females (565.86 ± 309.92 mGy·cm, 7.92 ± 4.34 mSv), while females had higher CTDIvol (15.58 ± 17.46 mGy) and SSDE (16.36 ± 18.33 mGy) than males (12.20 ± 5.64 mGy, 12.81 ± 5.92 mGy). Mann–Whitney U tests confirmed non-significance for DLP (U = 1227, *p* = 0.526), CTDIvol (U = 1090, *p* = 0.717), effective dose (U = 1225, *p* = 0.531), and SSDE (U = 1088, *p* = 0.722). These results indicate that protocol adjustments mitigate gender-specific variations. Figure 5 further depicts CTDIvol distribution patterns across BMI categories and gender.

### 3.5. Machine-Learning Models for Dose Prediction

Linear regression (baseline), Random Forest Regression, and Gradient Boosting Machines were developed to predict DLP and CTDIvol, using features including age, gender (encoded), BMI, kVp, mAs, pitch, and scan length. Features were normalized via Min-Max scaling, and selection via correlation analysis prioritized BMI, weight, mAs, and kVp (all |r| > 0.20, *p* < 0.05). The dataset was split into 70/15/15 (train/validation/test), with 5-fold cross-validation and a grid search over hyperparameters. For DLP prediction, Random Forest performed best (test set: MAE = 87.66 mGy·cm, RMSE = 112.34 mGy·cm, R^2^ = 0.77), followed by Gradient Boosting (MAE = 95.20, RMSE = 120.50, R^2^ = 0.74) and Linear Regression (MAE = 110.45, RMSE = 135.80, R^2^ = 0.68). For CTDIvol, Gradient Boosting excelled (MAE = 2.79 mGy, RMSE = 3.50 mGy, R^2^ = 0.79), with Random Forest (MAE = 3.10, RMSE = 3.85, R^2^ = 0.76) and Linear Regression (MAE = 3.85, RMSE = 4.60, R^2^ = 0.70). Feature importance (from Random Forest) highlighted BMI (0.38), mAs (0.32), weight (0.15), and kVp (0.10) as key predictors. These models generate a reference feature set for AI-driven protocol optimization, with Random Forest recommended for DLP due to its ability to handle non-linearities. The comparative performance of the developed models is summarised in Table 3.

## 4. Discussion

This study established local DRLs for adult chest CT by calculating the 75th percentile of CTDIvol and DLP within defined BMI categories. DRLs increased consistently with BMI, from 444.95 mGy·cm and 9.60 mGy in underweight patients to 931.60 mGy·cm and 20.25 mGy in obese patients. The differences in DLP and CTDIvol across BMI groups were statistically significant (*p* < 0.001), confirming that the required tube-output scales with patient habitus. These results demonstrate that a single, non-stratified “adult chest CT DRL” is clinically unsound. A universal threshold risks tolerating unnecessary exposure in smaller patients and, at the same time, labelling clinically necessary higher exposure in larger patients as excessive. BMI-stratified DRLs avoid both failure modes by providing habitus-appropriate investigation levels rather than a single pooled benchmark. Critically, these values should not be interpreted as hard dose limits. They function as escalation triggers: if a planned examination is expected to exceed the DRL for that patient’s BMI category, the protocol requires justification or adjustment before exposure. This reframes DRLs from a retrospective audit tool into an active, prescan safety control embedded in the daily workflow.

DLP showed moderate positive correlations with BMI and weight (r ≈ 0.54–0.56, *p* < 0.001), indicating that body habitus is the primary driver of delivered radiation output in routine chest CT. CTDIvol also increased with BMI and weight, though with slightly weaker correlation. Age demonstrated only a weak association with dose, and height was not clinically useful as a predictor. These findings indicate that dose modulation in current practice is fundamentally governed by patient size rather than age, height, or sex. Consistent with this, there were no statistically significant differences between males and females in either CTDIvol or DLP (*p* > 0.5), supporting the conclusion that sex-specific DRLs are unnecessary once BMI is accounted for.

These correlations have direct procedural consequences. First, BMI and weight can be used prospectively to inform tube current modulation, kV selection, and reconstruction strategy before scanning, rather than being considered only after scanning during periodic audit. Second, they can be embedded into protocol decision support. For example, if a high-BMI patient is predicted to receive a DLP that substantially exceeds the obese-group DRL, the system can alert the technologist to reassess exposure parameters in advance. In other words, patient factors become inputs into active dose control rather than passive descriptors of dose.

Traditional DRLs, defined as the 75th percentile of CTDIvol or DLP for a given anatomical protocol, were designed to benchmark practice and identify outliers for audit. However, conventional DRLs assume a homogeneous “standard adult,” an assumption that fails in populations with a wide BMI distribution. Recent optimization work has argued for weight-banded or habitus-adjusted DRLs to improve interpretability, fairness, and clinical utility. The present study operationalizes that direction in adult chest CT by providing explicit BMI-stratified DRLs and demonstrating statistically separable dose behavior between BMI groups.

This has two implications for interpreting dose relative to “high” values reported elsewhere. First, higher absolute DLP in obese patients should not automatically be classified as unjustified overexposure; it should be interpreted relative to an obese-specific DRL. Second, elevated DLP in a low-BMI patient is more concerning and should immediately trigger protocol review. This contextualized interpretation is more defensible clinically than comparing all patients to a single pooled reference.

The correlation structure in this dataset is consistent with prior observations that BMI, weight, and scan geometry dominate dose behavior. At the same time, demographic variables such as sex do not independently justify different reference levels once habitus is controlled. The present work extends that literature by linking these clinical observations to predictive modelling. Machine-learning models (including ensemble regressors such as Random Forest and Gradient Boosting) trained on routinely available variables (BMI, weight, kVp, mAs, scan length) predicted CTDIvol and DLP with high performance (R^2^ approaching ~0.8). This moves beyond descriptive statements such as “larger patients receive higher doses” and demonstrates that the expected dose for an individual patient under a proposed protocol can be forecast before acquisition.

The dependency DLP ≈ CTDIvol × L explains the small but systematic performance differences between the two targets in Table 3. CTDIvol is chiefly governed by protocol and habitus. In contrast, DLP embeds an additional, partly independent source of variance scan length, which introduces “human-in-the-loop” variability from technologist coverage selection and series structure. Consequently, prediction error in determinants of CTDIvol is propagated through multiplication by a variable *L*, typically yielding higher MAE/RMSE for DLP than for CTDIvol at comparable R^2^. To avoid information leakage, each target was modelled independently: when predicting DLP, CTDIvol was not included as a predictor (and vice versa); DLP models included scan length, whereas CTDIvol models did not include DLP. Thus, reported performance reflects clinically actionable features (habitus, planned parameters, scan length) rather than a trivial identity.

Extension to screening and pseudo-screening CT. For low-dose screening (and opportunistic pseudo-screening), the workflow would surface predicted CTDIvol/DLP against screening-specific BMI bands and trigger a single prescan adjustment (e.g., reduced mAs or DL reconstruction) only when exceeding the envelope. Because screening programs prioritize very low dose and consistent image quality, we recommend incorporating size-specific metrics (SSDE)**,** program-level QA dashboards, and periodic drift monitoring. This preserves the benefits of AI-assisted, patient-centered optimization while aligning with screening governance requirements.

While models here are data-driven, hybrid, physics-informed constraints (e.g., tube-output scaling, size-to-dose mappings, scanner-specific calibration layers) may improve interpretability and cross-scanner robustness. Future work includes multi-vendor validation with these constraints to assess transportability and operational fit. While models here are data-driven, hybrid, physics-informed constraints (e.g., tube-output scaling, size-to-dose mappings, scanner-specific calibration layers) may improve interpretability and cross-scanner robustness. Future work includes multi-vendor validation with these constraints to assess transportability and operational fit.

## 5. Limitations

This single-center, single-vendor cohort limits generalizability across scanners, exposure-control implementations, and protocol philosophies. Representation at BMI extremes was sparse, increasing percentile uncertainty for underweight and morbidly obese groups. Models were trained and tested on institutional data only, so performance may reflect site-specific regularities; no external validation was performed. Activity-/diagnostic-based image-quality assessment was not available in this retrospective dataset and was therefore not included; accordingly, a lower predicted dose cannot be assumed to remain clinically acceptable. Prospective, multi-center work will incorporate task-based/reader studies and phantom-based metrics to link dose reduction with diagnostic adequacy. Operational variability (e.g., technologist-selected coverage affecting scan length) was only partially captured by available features.

## 6. Conclusions

This study delivers a practical framework that pairs BMI-stratified DRLs with supervised prediction of CTDIvol and DLP from routinely available variables, enabling prospective, patient-centered dose governance in chest CT. By comparing prescan model predictions with BMI-matched DRLs at the console, teams can adjust kVp/mAs and reconstruction parameters before exposure, aligning radiation output with habitus while safeguarding diagnostic utility. The approach moves dose management from retrospective audit to real-time decision support and provides a transferable template for service-level optimization. Priorities now are multi-center, multi-vendor validation and integration of task-based image quality to ensure that dose reductions translate into safe, diagnostically adequate imaging.

## Figures and Tables

**Table 1:** Descriptive statistics for dose metrics and patient characteristics (*n* = 100).

	Mean	SD	Min	Max	25%	50%	75%
DLP (mGy·cm)	575.47	280.01	108.30	1374.20	367.90	514.30	753.60
CTDIvol (mGy)	13.59	12.05	2.79	113.00	8.00	11.40	16.00
Scan Length (cm)	48.01	16.21	9.64	75.15	36.57	40.19	66.04
Height (cm)	164.52	9.18	142.00	186.00	159.00	164.00	171.00
Weight (kg)	72.26	20.68	33.00	161.00	57.30	70.00	82.33
Age (years)	46.79	20.17	15.00	91.00	30.00	48.00	62.00

**Table 2:** BMI-stratified DRLs (75th percentile) for key dose metrics.

BMI Category	Total	DLP (mGy·cm)	CTDIvol (mGy)	Effective Dose (mSv)	SSDE (mGy)
Underweight	12	444.95	9.60	6.23	10.08
Normal	38	513.00	11.55	7.18	12.11
Overweight	30	756.08	14.65	10.59	15.38
Obese	20	931.60	20.25	13.04	21.26

**Table 3:** Performance on the held-out test set. Each target was modelled independently (no cross-use of CTDIvol/DLP). DLP models included scan length; CTDIvol models did not include DLP.

Model	Target	MAE	RMSE	R^2^
Linear Regression	DLP	110.45	135.80	0.68
Random Forest		87.66	112.34	0.77
Gradient Boosting		95.20	120.50	0.74
Linear Regression	CTDIvol	3.85	4.60	0.70
Random Forest		3.10	3.85	0.76
Gradient Boosting		2.79	3.50	0.79

## Data Availability

Upon request, the main author will provide all related case data.

## Abbreviations

AI	Artificial intelligence
BMI	Body mass index
CTDIvol	Volume CT dose index
DLP	Dose–length product
DRL	Diagnostic reference level
